# Investigation of Gene Expressions of Myeloma Cells in the Bone Marrow of Multiple Myeloma Patients by Transcriptome Analysis

**DOI:** 10.4274/balkanmedj.2018.0356

**Published:** 2019-01-01

**Authors:** Melda Sarıman, Neslihan Abacı, Sema Sırma Ekmekçi, Aris Çakiris, Ferda Perçin Paçal, Duran Üstek, Mesut Ayer, Mustafa Nuri Yenerel, Sevgi Beşışık, Kıvanç Çefle, Şükrü Palandüz, Şükrü Öztürk

**Affiliations:** 1 Department of Genetics, İstanbul University, Aziz Sancar Experimental Medical Research Institute, İstanbul, Turkey; 2 Clinic of Hematology, İstanbul Haseki Training and Research Hospital, İstanbul, Turkey; 3 Department of Hematology, İstanbul Universiy İstanbul School of Medicine, İstanbul, Turkey; 4 Department of Internal Medicine, İstanbul Universiy İstanbul School of Medicine, İstanbul, Turkey; 5 Department of Internal Medicine, Division of Medical Genetics, İstanbul Universiy İstanbul School of Medicine, İstanbul, Turkey

**Keywords:** Flow cytometry, gene expresion, multiple myeloma, plasma cell dyscrasias, transcriptome analyses

## Abstract

**Background:**

Multiple myeloma is a plasma cell dyscrasia characterized by transformation of B cells into malignant cells. Although there are data regarding the molecular pathology of multiple myeloma, the molecular mechanisms of the disease have not been fully elucidated.

**Aims:**

To investigate the gene expression profiles in bone marrow myeloma cells via RNA-sequencing technology.

**Study Design:**

Cell study.

**Methods:**

Myeloma cells from four patients with untreated multiple myeloma and B cells from the bone marrow of four healthy donors were sorted using a FACSAria II flow cytometer. The patient pool of myeloma cells and the control pool of B cells were the two comparative groups. A transcriptome analysis was performed and the results were analyzed using bioinformatics tools.

**Results:**

In total, 18.806 transcripts (94.4%) were detected in the pooled multiple myeloma patient cells. A total of 992 regions were detected as new exon candidates or alternative splicing regions. In addition, 490 mutations (deletions or insertions), 1.397 single nucleotide variations, 415 fusion transcripts, 132 frameshift mutations, and 983 fusions, which were reported before in the National Center for Biotechnology Information, were detected with unknown functions in patients. A total of 35.268 transcripts were obtained (71%) (25.355 transcripts were defined previously) in the control pool. In this preliminary study, the first 50 genes were analyzed with the MSigDB, Enrichr, and Panther gene set enrichment analysis programs. The molecular functions, cellular components, pathways, and biological processes of the genes were obtained and statistical values were determined using bioinformatics tools and are presented as a supplemental file.

**Conclusion:**

*EEF1G, ITM2C, FTL, CLPTM1L*, and *CYBA* are identified as possible candidate genes associated with myelomagenesis.

Multiple myeloma (MM) is a cancer of plasma cells in which abnormal types of immunoglobulins are produced that can be measured in blood and urine (1). MM is a clonal B cell malignancy characterized by a clinical pentad in the bone marrow (2). According to the National Cancer Institute Surveillance, Epidemiology, and Results Program in 2017, 30,280 new cases of MM were diagnosed, and 12,590 deaths occurred in the United States (3).

The etiology of MM depends on many factors, such as the environment, chemical agents, viruses, and genetic factors (4). MM is caused by several molecular mechanisms, such as activation of oncogenes, genomic instability, and chromosome abnormalities (5). Accumulated plasma cells in the bone marrow of patients with MM are identified by surface membrane antigens, such as CD38, CD138, CD56, CD117, and CD33. Although most myeloma cells typically express CD38(+), CD138(+), CD56(+), and CD19(-), these cells may indicate genetic heterogeneity (6). Next generation sequencing is evolving rapidly, and RNA-seq has become a widely used tool for understanding the molecular mechanisms of human cancers. The transcriptome is the complete set of transcripts in a cell that allows for the identification of exons and alternative splicing/isoforms and novel RNAs. Massive parallel sequencing platforms are used to measure the differential expression of transcripts (7).

In the present study, we provide comprehensive insight into the transcriptomes of patients with MM and healthy donors. Many of the genes were determined in transcriptome profiles of pooled myeloma cells and were determined to play a role in MM pathogenesis via intracellular pathways, gene expression patterns, biological functions, and protein-protein interactions using *in silico* tools. Our transcriptomic profile obtained data to evaluate differential expression of all transcripts, alternative new splicing variants, mutations, and fusion genes. These results will contribute to the understanding of myeloma pathogenesis and provide valuable information for prognostication and new therapies.

## MATERIALS AND METHODS

### Sample collection

This study was approved (2010/108-28) by the Ethics Committee of İstanbul University Faculty of Medicine. Written informed consent Helsinki Declaration and ethics committee documents was obtained from all patients and healthy donors. We performed RNA-seq using the Ion Torrent Personal Genome Machine (PGM) platform to compare the transcriptome profiles of four newly diagnosed patients with untreated MM and four healthy donors. Bone marrow was aspirated from the hip bones of all patients and donors. The bone marrow samples were subjected to Ficoll gradient centrifugation (1.077 g/mL Ficoll), and the mononuclear cells were collected. The viability and absolute cell counts were determined by the Vi-CELL™ XR Cell Counter (Beckman Coulter, Brea, CA, USA).

### Fluorescence-activated cell sorting

**Figure 1:**
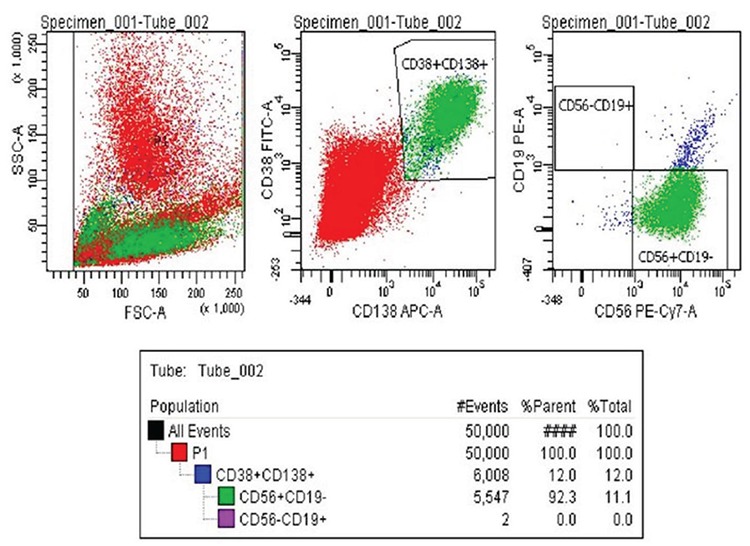
Flow cytometry results of malignant B cells from bone marrow of a patient with Multiple myeloma. First, myeloma cells were gated by using specific cell surface markers that were CD138^+^ and CD38^+^ by determining forward and side light scattering characteristics on the FACSAria II Cell Sorter (Becton Dickinson, San Jose, CA, USA). Then sorted malignant Multiple myeloma cells using with cell sorting by the cell surface markers CD56+, CD19- according to the FACSAria II Cell Sorter.

**Figure 2:**
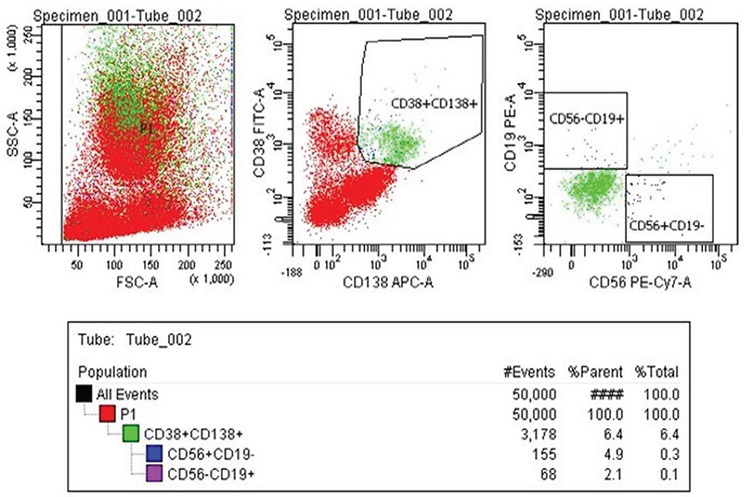
Bone marrow B cell flow cytometry results from a healthy donor. First, B cells were gated by using specific cell surface markers that were CD138^+^ and CD38^+^ by determining forward and side light scattering characteristics on the FACSAria II Cell Sorter (Becton Dickinson, San Jose, CA, USA). Then sorted B cells using with cell sorting by the cell surface markers CD56^+^, CD19^+^ according to the FACSAria II Cell Sorter.

Myeloma cells (CD38+, CD138+, CD19-, and CD56+) and healthy B cells (CD38+, CD138+, CD19+, and CD56-) were selected from bone marrow mononuclear cells using a gating strategy by simultaneously specifying cell surface markers, and by determining forward and side light scattering characteristics on the FACSAria II Cell Sorter (Becton Dickinson, San Jose, CA, USA) (Figures 1, 2). The antibodies used for activating fluorescence and cell sorting were CD138/SYNDECAN-1 (cat: 347216) allophycocyanin, CD38 (cat: 340909) fluorescein isothiocyanate, CD19 (cat: 345777) phycoerythrin, CD56 (cat: 557747), and phycoerythrin cyanin (Becton Dickinson). The cells were passed through a 100 µm nozzle tip at a speed of approximately 50,000 events per sec. The images were taken and the analysis was performed using FACS Diva Software 6.1.2. The sorted cells were frozen for RNA isolation.

### RNA isolation

RNA was extracted from the sorted cells using the PureLink RNA Microkit (cat: 12183_016; Invitrogen, Carlsbad, CA, USA). Before proceeding to rRNA depletion, the quantity and quality of total RNA was evaluated using the RNA 6000 Pico kit on the Agilent 2100 Bioanalyzer (Agilent Technologies, Anaheim, CA, USA). After checking the quantity and quality of the RNA, we pooled the RNA samples from the four untreated MM patients and four healthy donors. The workflow of the study is summarized in Table 1.

### rRNA depletion

rRNA depletion was performed using the Eukaryotic Ribominus kit (cat no: A10837_2/A10837_08; Invitrogen). The quantity and quality of the mRNA was evaluated using the RNA 6000 Pico kit on Agilent 2100 Bioanalyzer.

### Library preparation and RNA sequencing

**Figure 3:**
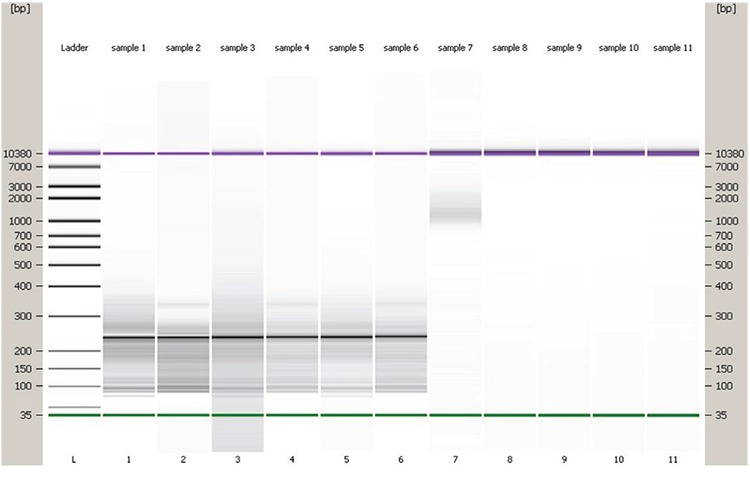
In Agilent Bioanalyzer gel-like image of cDNA. This image shows produced cDNA samples quality controls. After library construction for the quality of the libraries was validated using the Agilent High Sensitivity DNA kit on the Agilent 2100 Bioanalyzer. DNA ladder (L), Lanes 1-3-5 (control cDNA library), the lanes 3-5 are ten-fold diluted sample 1. Lanels 2-4-6 (Multiple myeloma cDNA library). The lanes 4-6 are ten- fold diluted sample 2. Lane 7 (negative). Green lines indicate the low weight (35 base pairs) DNA ladder, Purple lines the high weight (10380 base pairs) DNA ladder.

The RNA-sequencing libraries were prepared from the pooled RNA of the patients and healthy donors. The libraries were constructed using the Ion Total RNA-seq V2 kit protocol for transcriptome profiling of low-input RNA samples (April 2011). The quality of the libraries was validated using the Agilent High Sensitivity DNA kit on the Agilent 2100 Bioanalyzer (Figure 3). The final library concentrations were calculated after validating the libraries, using the Ion PGM™ 200 Xpress Template kit (cat no: 4474280). Each library was amplified by Emulsion polymerase chain reaction of ion sphere particles (ISPs). These ISPs were recovered from the emulsion. The templated ISPs and unbound ISPs were determined by fluorometric assay on Qubit^®^ 2.0.

The Ion PGM™ system was cleaned and initialized before sequencing. Sequencing was accomplished using the Ion PGM™ 200 Sequencing v2 kit (cat: 4474004; Life Technologies, Carlsbad, CA, USA) according to the manufacturer’s instructions. Pooled samples were sequenced with a semiconductor-based sequencing system using the Ion 318™ Chip (lots: P30518.1, AA0150428, and AA0150429; Life Technologies).

### RNA-sequencing analysis

The pooled RNA from the patients with MM and that from the controls was subjected to massively parallel cDNA sequencing at İstanbul University Whole Genome Sequencing Laboratory Aziz Sancar Institute of Experimental Medicine. The sequencing data were analyzed with the PSSC Labs Big Data Server (Lake Forest, CA, USA). The quality of the raw sequencing data was checked and the data were preprocessed according to two criteria: adaptors and low quality sequences (readings with ambiguous N’s) were removed, and data with a quality score (Qscore) <30 was trimmed.

### Bioinformatics analysis

**Figure 4:**
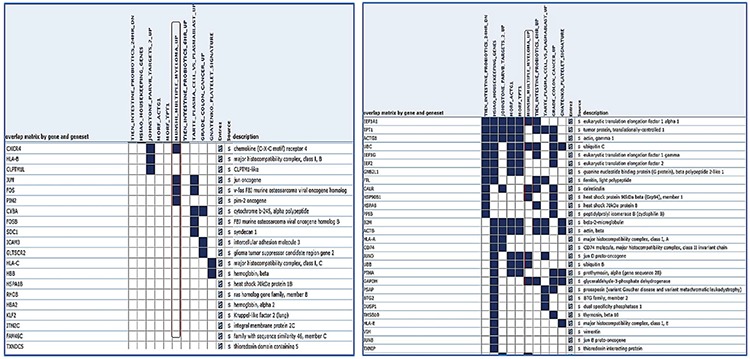
The first 50 highly expressed selected genes in the pooled Multiple myeloma cells results of the Gene Set Enrichment Analysis-MSigDB program. Summary figures displayed enriched gene sets with three major columns: gene names, the images of overlapping between gene and gene sets, descriptions of gene names. These 50 genes to compare with other gene set libraries subjected to the Gene Set Enrichment Analysis using MSigDB. Shown in the figure, 11 of these genes had increased expression in myeloma cells that significantly overlapped between *EEF1A1, UBC, UBB, CALR, CXCR4, JUND, FOS, PIM2, JUN, GAPDH*, and *HSP90B1* and were previously reported as upregulated in the Munshi Multiple myeloma dataset.

The trimmed sequencing reads were aligned to the UCSC human reference genome (build GRCh37/hg19) using TopHat v2.0.6, which incorporates Bowtie v0.12.8 software to build the alignment. The expression level for each transcript was normalized to the reads per kilobase of the exon model per million mapped reads (RPKM) (8). Cufflinks v2.0.2 was used to operate the original alignment file generated by tophat and the gene transfer format file for genome annotation to determine the difference between the expressed genes. The first 50 genes from the pooled MM RNA that were highly differentially expressed were analyzed using the Gene Set Enrichment Analysis (GSEA) program (Figure 4) and also demonstrated protein interactions.

## RESULTS

The cDNA libraries from the patients with MM and the control group were subjected to massively parallel transcriptome sequencing. Of the 18,806 total transcripts obtained by the transcriptome analysis, 17,760 were reported previously in pooled patients with MM. These transcripts were used in the downstream analysis.

**Figure 5:**
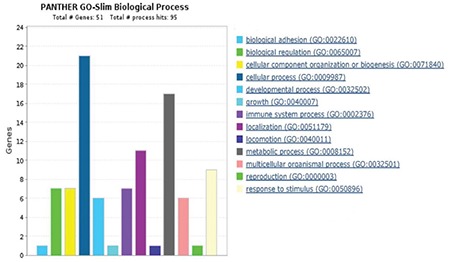
Biological process analysis of the first 50 highly expressed genes according to the PANTHER program: The highest rated biological process is cellular process analyzed in bar view. It is next to the color indicator that identifies the respective GO terms and numbers according to PANTHER.

**Figure 6:**
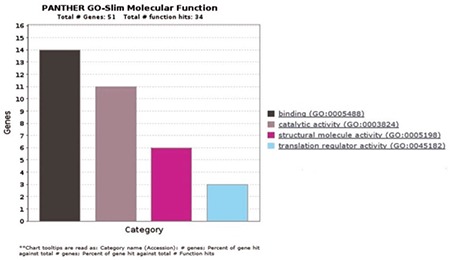
Molecular function analysis of the first 50 highly expressed genes according to the PANTHER program: The highest rated molecular functions is binding analyzed in bar view. It is next to the color indicator that identifies the respective GO terms and numbers according to PANTHER.

**Figure 7:**
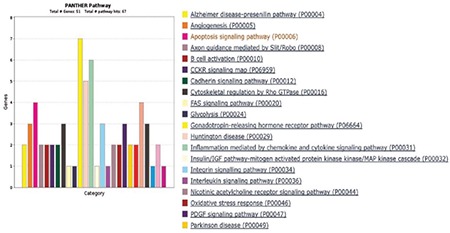
Pathway analysis of the first 50 highly expressed genes according to the PANTHER program. Results of PANTHER colorful bars view show us several myeloma pathways associated with especially the B cell activation, inflammation mediated by chemokine and cytokine signaling pathway and apoptosis signaling pathway.

**Figure 8:**
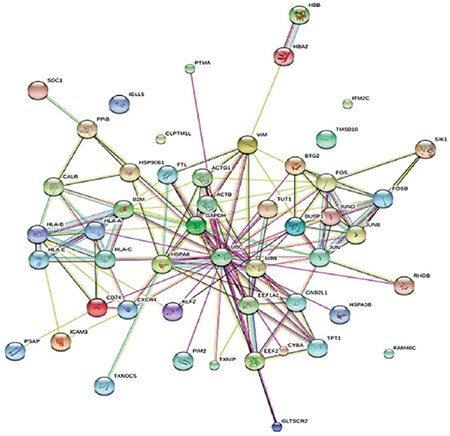
Protein-protein interactions network of between the first 50 highly differentially expressed genes in myeloma cells. The network was constructed using the STRING database (http://string-db.org).

In total, 992 regions were detected and were candidates for new exons or alternative splicing regions. In addition, 490 deletions or insertions and 1.397 single nucleotide variations were detected; 415 fusion transcripts were defined. A total of 983 fusions, which were reported before in National Center for Biotechnology Information, were detected with unknown functions. In total, 132 frame shift mutations were identified in pooled patients with MM; 35,268 transcripts were obtained (71%) (25,355 transcripts were defined previously) in the control pool. Qscore values of about 35% on average for each chromosome were calculated. We measured the transcript values and identified the differentially expressed genes between the two groups using Cuffdiff/Cuflink. In total, we detected 12,453 expressed genes by calculating reads per kilobase million (RPKM) values and analyzed the data from the first 50 highly expressed selected genes in the pooled MM cells (Table 2) and compared the expression levels with the controls (Table 3). The eukaryotic elongation factor 2 (*EEF2*) gene was the most significantly expressed gene among the MM and normal cells according to our RPKM results. Our analysis included the majority of annotated human genes. The analysis of the whole transcriptome data revealed different expression levels of several genes, such as *JAK1*, *JAK2*, *JAK3*, *RAF*, *IL6R*, *NCAM (CD56)*, *WHSC1*, *MCL1*, *BCL2,* and *IGF1*, which showed myeloma pathogenesis as reported previously. These 50 genes were subjected to the GSEA using MSigDB. As a result, 11 of these genes had increased expression in plasma cells from patients with MM that significantly overlapped between *EEF1A1*, *UBC*, *UBB*, *CALR*, *CXCR4*, *JUND*, *FOS*, *PIM2*, *JUN*, *GAPDH*, and *HSP90B1* and were previously reported as upregulated in the Munshi_multiple _myeloma data set (Figure 4). The biological functions, molecular processes, and pathways of these genes were determined using the online tool Panther-GO (Figures 5,6,7). These genes were also analyzed by the String v9.0 program to demonstrate protein-protein interactions (Figure 8). We performed the bioinformatics calculations using the Enrichr GSEA program. Computational bioinformatics was used to explore the deep relationships between the genes in the annotated gene sets and between other data sets. We investigated and visualized the overlap between our data sets within the Enrichr program to compare with other web-server tools and resources that serve gene set libraries. The molecular functions, cellular components, pathways, and biological processes are presented as a supplemental file.

## DISCUSSION

Decades of scientific research data backed up with array technologies and next generation sequencing technologies have shown us two important contributors to MM pathogenesis. One is the interactions between myeloma cells and the microenvironment and the other is malignant clone genetic transformation (9,10). In this study, we purified and directly sorted myeloma cells. Multicolor flow cytometry is a sensitive method to analyze plasma cell immunophenotypes and identify normal and neoplastic plasma cell populations. In our study, the surface markers of malignant B cells obtained from the literature were compared with those of the MM group with CD38(+), 138(+), CD56(+), CD19(-) B cell separation of the control group using CD38(+), 138(+), CD56(-), CD19(+) surface markers by multicolor flow cytometry (11). The transcriptome patterns of the first 50 highly expressed genes in Figure 4 and in those in Figures 5,6,7 were analyzed, and a pathway network was constructed to better understand the relationships among them. Technologies must be developed to identify related genes that show potential to play a role in transforming normal cells into myeloma cells. GSEA MSigD identified genes were included in previously known and myelomagenesis signaling related pathways. In addition, the families of these genes were analyzed with the same program. According to the results, *JUN* was in the oncogene family; *FOSB*, *JUN*, *JUNB*, *JUND*, and *KLF *were in the transcription factor family; *PIM2* and *SIK1* were in the protein kinase family; and *CXCR4*, *CD74,* *ICAM3*, and *SDC1* were cell differentiation markers. In particular, the first 50 genes supported that the ubuquitin genes, such as *UBB*, *UBC*, *EEF2*, were related to the pathogenesis of MM. The ubiquitin cascade system is a central contributor to cellular processes that regulate protein stability, trafficking, and activation (12,13). Proteosome inhibitors have been used for many years as a basic therapeutic strategy for treating MM and have been developed as antimyeloma therapy by focusing on this ubiquitin proteosome cascade (14). Losada et al. (15) reported that plitidepsin has successfully concluded a phase-III clinical trial for MM. Antitumor activity was achieved by targeting plitidepsin to *EEF1A2*. *EEF1A2* has proto-oncogenic activity, and it has been reported to be abnormally expressed in many human tumors including MM. In our study, the *EEF2*, *EEF1G*, and *EEF1A1* genes were overexpressed and may be responsible for inhibiting apoptosis and controlling unfolded protein degradation by proteasomes similar to the EEF1G gene. Prosaposin is a lysosomal protein that has pleiotropic growth factor activity. It is known to be related to the growth of breast cancer and to increase ER levels through the mitogen activated protein kinase (MAPK) pathway. In addition to gallbladder cancer, it operates as a biomarker and promotes increased degradation of ceramides, ensuring a survival advantage to cancer cells (16). Starlets et al. (17) reported that a cell surface molecule expressed on B cells binds CD74 to the macrophage migration-inhibition factor, activating CD74; thus, initiating a survival pathway. The humanized anti-CD74 monoclonal antibody acts as a potential therapeutic agent by exhibiting cell proliferation activity in MM (18). Our findings show that CD74 is strongly expressed in myeloma cells compared to healthy cells and has a role in the oncogenic process of cell proliferation and survival. Prosaposin is a protein encoded by the *PSAP* gene that interacts with CD74 and may play a role in MM carcinogenesis (Figure 8). Despite advances in the understanding of MM pathogenesis, the molecular pathways underlying the development of MM are still unknown. Our functional and pathway enrichment analysis proposed that major histocompatibility complex class I molecules called human leukocyte antigens (HLA-A, HLA-B, HLA-C, and HLA-E) are involved. Known as antigen processing and presenting machinery (AMP), these molecules are important for cell survival, cell cycle progression, and inhibition of apoptosis. Defects in the AMP lead to immune escape and continuity by cancer. As a result, it enables malignant transformation of cells.

The enriched pathways for these upregulated genes are involved in peptide transport from the cytosol into the endoplasmic reticulum, antigen processing, peptide trimming, and assembly of the major histocompatibility complex class I loading complex (19). Leone et al. (20) compared the expression levels of calnexin, calreticulin, tapasin, and *ERp57* genes in premalignant plasma cells obtained from patients with monoclonal gammopathy of undetermined significance, those with MM, and normal plasma cells from healthy donors and showed that these levels are higher in patients with monoclonal gammopathy of undetermined significance and MM. It has been documented in cell lines from primary cells and various tumors, particularly MM, that *TAP1* and/or *TAP2* mRNA and protein levels are not detectable in small quantities. Defects in *TAP* genes play a role in the development of hematological malignancies (21). Our results included a mutated *TAP* gene (data not shown). Another related gene is calreticulin. This gene produces a calcium-binding protein that is a major component of the endoplasmic reticulum and has been shown in various cell types to be involved in regulating calcium homeostasis, as a ligand for integrins, and as a component of phagocytic synapses (22). Upregulation of the calreticulin gene is an adverse prognostic factor as the dominant pro-phagocytic signal in diverse tumors and is correlated with increased CD47 expression in cancer cells (23). The cisplatin resistance-related protein CRR9p (*CLPTM1L*) gene is overexpressed in lung cancer and knockdown of this gene prevents 95-D lung cancer cells from migrating and invading (24). Although the function of this gene is largely unknown, high expression levels of *CLPTM1L* have been observed in many cancers, and it is linked with cisplatin-induced apoptosis (25). Another study demonstrated that blocking *CLPTM1L* with interfering RNA inhibits lung tumorigenesis induced by K-RAS. That study suggested that *CLPTM1L* interacts with PI3 kinase and has a major role in *RAS*-induced *AKT* phosphorylation (26). Our results suggest that the *CLPTM1L* gene has a higher RPKM value in myeloma cells than in healthy donor cells. Cytokines and growth factors activate the phosphoinositide 3-kinase/AKT signaling cascade, creating life signals for myeloma cells by inhibiting apoptosis in MM. The RAS-MAPK pathway provides for proliferation of myeloma cells (27). The results of other studies on this gene are particularly related to PI3-K/AKT cascades, indicating that it should be considered a plausible candidate gene. Dytfell et al. reported that increased *TXNDC5* expression in plasma cells and serum is related to a poor response to bortezomid-based therapy in patients with newly diagnosed MM and in those with relapsed MM. Understanding MM biology and identifying drug-resistance biomarkers are vital to enable the development of individualized treatments. Proteomic signature results indicate that *TXNDC5*, which is a member of the protein disulfide isomerase family, shows increased expression and is involved in protection against oxidative stress and plays a major role in bortezomib treatment (28). Using in silico tools, we identified significant overlaps between *EEF1A1*, *UBC,* *UBB*, *CALR*, *CXCR4*, *JUND*, *FOS*, *PIM2*, *JUN*, *GAPDH*, and *HSP90B1*, which were previously reported to be upregulated in MM according to the Munıshı_ MM data set (Figure 4). The Panther program revealed the results of molecular features, biological functions, and related pathways of the first 50 genes with the highest RPKM values. As we compared our findings with the results from other studies, we saw that biologic adhesion, biological processes, and metabolic processes were the same as these previous studies (Figures 5,6,7). Analyzing these genes using the String program may lead to further functional studies on protein-protein interactions (Figure 8). This transcriptome analysis of MM was performed for the first time in a Turkish population. As results, we determined some variations and different mRNA expression patterns in some of the previously reported genes particularly those in the ubiqutin-proteosomal pathway.

In conclusion, *EEF1G*, *ITM2C*, *FTL*, *CLPTM1L*, and *CYBA* are possible candidate genes associated with myelomagenesis.

## Figures and Tables

**Table 1:**
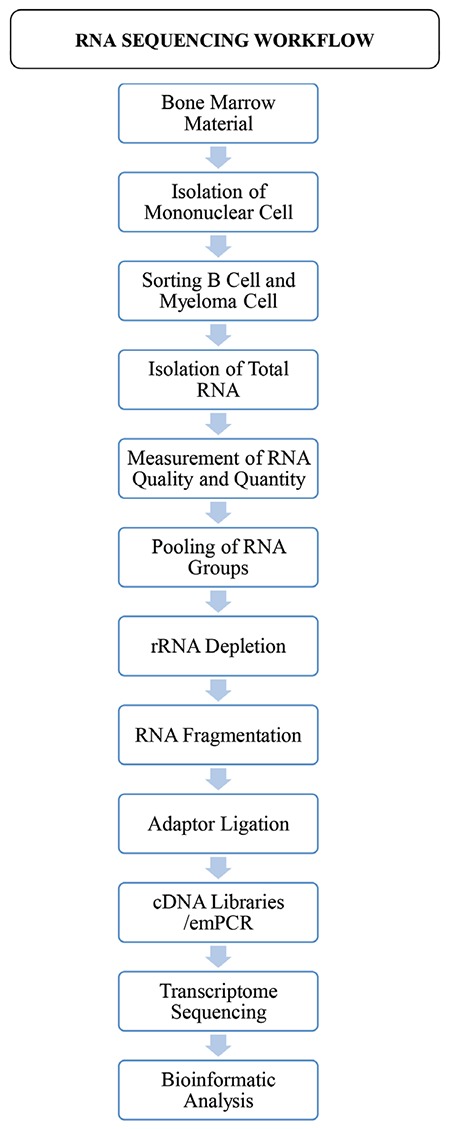
RNA-sequencing workflowcells

**Table 2:**
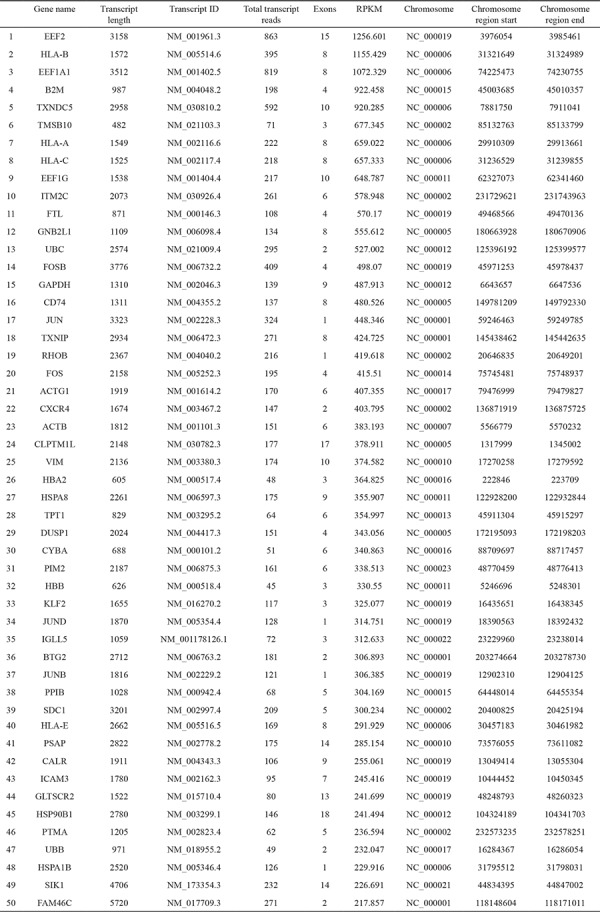
Reads per kilobase of the exon model per million mapped reads values of the first 50 selected genes highly expressed in pooled in multiple myeloma cells

**Table 3:**
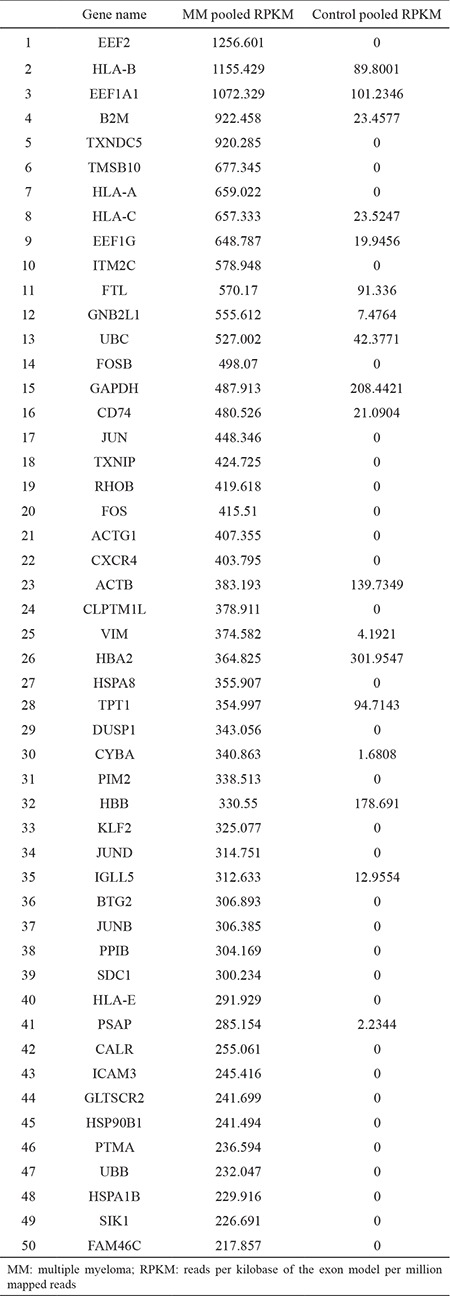
Comparison of the first 50 genes which have highest reads per kilobase of the exon model per million mapped reads value in pooled multiple myeloma with pooled healthy control
